# Identification of Retinal Ganglion Cells and Their Projections Involved in Central Transmission of Information about Upward and Downward Image Motion

**DOI:** 10.1371/journal.pone.0004320

**Published:** 2009-01-29

**Authors:** Keisuke Yonehara, Hiroshi Ishikane, Hiraki Sakuta, Takafumi Shintani, Kayo Nakamura-Yonehara, Nilton L. Kamiji, Shiro Usui, Masaharu Noda

**Affiliations:** 1 Division of Molecular Neurobiology, National Institute for Basic Biology, Okazaki, Japan; 2 School of Life Science, The Graduate University for Advanced Studies, Okazaki, Japan; 3 Laboratory for Neuroinformatics, RIKEN Brain Science Institute, Wako, Saitama, Japan; 4 Department of Psychology, Graduate School of the Humanities, Senshu University, Kawasaki, Kanagawa, Japan; Smithsonian Institution, United States of America

## Abstract

The direction of image motion is coded by direction-selective (DS) ganglion cells in the retina. Particularly, the ON DS ganglion cells project their axons specifically to terminal nuclei of the accessory optic system (AOS) responsible for optokinetic reflex (OKR). We recently generated a knock-in mouse in which SPIG1 (SPARC-related protein containing immunoglobulin domains 1)-expressing cells are visualized with GFP, and found that retinal ganglion cells projecting to the medial terminal nucleus (MTN), the principal nucleus of the AOS, are comprised of SPIG1^+^ and SPIG1^−^ ganglion cells distributed in distinct mosaic patterns in the retina. Here we examined light responses of these two subtypes of MTN-projecting cells by targeted electrophysiological recordings. SPIG1^+^ and SPIG1^−^ ganglion cells respond preferentially to upward motion and downward motion, respectively, in the visual field. The direction selectivity of SPIG1^+^ ganglion cells develops normally in dark-reared mice. The MTN neurons are activated by optokinetic stimuli only of the vertical motion as shown by Fos expression analysis. Combination of genetic labeling and conventional retrograde labeling revealed that axons of SPIG1^+^ and SPIG1^−^ ganglion cells project to the MTN via different pathways. The axon terminals of the two subtypes are organized into discrete clusters in the MTN. These results suggest that information about upward and downward image motion transmitted by distinct ON DS cells is separately processed in the MTN, if not independently. Our findings provide insights into the neural mechanisms of OKR, how information about the direction of image motion is deciphered by the AOS.

## Introduction

Visual information is segregated into modalities such as color, form, and motion at the level of the retina [1]. Retinal ganglion cells comprise more than a dozen different types with distinct structure and function [2], [3], and different modalities are conveyed parallel to the different sites in the brain by the axons of different types of retinal ganglion cells [4]. Each ganglion cell type develops dendrites in a specific stratum in the inner plexiform layer to receive distinct information about visual world from distinct set of amacrine cells and bipolar cells [5].

Two types of direction-selective (DS) ganglion cells, ON DS ganglion cells and ON-OFF DS ganglion cells, present in the retina of many vertebrate species [6], are known to code the direction of image motion [7], [8]. ON DS cells respond to bright objects moving at slow speeds, whereas ON-OFF DS cells respond to both bright and dark objects moving over a broad range of speeds. It is known that ON DS cells comprise three physiological subtypes distinguished by their preferred directions, each corresponding to upward, downward, or temporal-to-nasal motion in the visual field [9]. On the other hand, ON-OFF DS cells are of four subtypes; their preferred directions are orthogonal and correspond roughly to upward, downward, temporal-to-nasal, and nasal-to-temporal motion [10]. Numerous studies have been undertaken to understand the cellular mechanisms for direction selectivity in the retina [11], [12] since they were discovered, for the first time, in the rabbit retina [7]. To generate a direction-selective response, the DS cells need to collect inhibitory synapses selectively from a subset of starburst processes, which mostly point in the DS cell's null direction [13]. The mechanism underlying the detection of motion direction is presumably common to ON and ON-OFF types [14], [15], however, how direction-selective retinal circuitry is established during development still remains unknown. Identification of molecular markers for a single physiological subtype of DS cells would be useful to study its developmental mechanism.

The accessory optic system (AOS) nuclei are suggested to be the first post-retinal relay station in the pathway mediating the vertical and horizontal optokinetic reflex (OKR), a class of compensatory eye movements that serve the functional purpose of stabilizing images on the retina during self-motion and/or motion of the visual surround [16]. The AOS is therefore needed for proper visual acuity [17] and also velocity discrimination [18]. The neurons in the AOS nuclei show speed and direction selectivity [19], which are similar to those of ON DS cells as demonstrated in rabbits [20]: The neurons in the AOS nuclei respond well to bright objects moving at slow speeds, and their preferred directions roughly correspond to upward, downward, or temporal-to-nasal motion in the visual field [19]. These observations, together with the morphological appearance of AOS-projecting cells [21], [22], suggested that the retinal input to the AOS is largely from the ON DS cells [19]. On the other hand, ON-OFF DS cells supposedly project to the dorsal lateral geniculate nucleus [23] and superior colliculus [24]. Elimination of starburst amacrine cells not only abolishes direction-selective responses of retinal ganglion cells but also severely impairs OKR, suggesting that DS cells mediate OKR [25]. However, the extent to which ON DS cells and ON-OFF DS cells contribute to the OKR respectively is largely unknown. Thus, the AOS provides a unique opportunity to examine the correlation between the activity of single retinal ganglion cell type and a well characterized animal behavior.

In most mammalian species, the AOS consists of two sets of optic fibers, the inferior fasciculus of the accessory optic tract (AOT-IF) and superior fasciculus of the AOT (AOT-SF); and three terminal nuclei, medial terminal nucleus (MTN), lateral terminal nucleus (LTN), and dorsal terminal nucleus (DTN) [26]. The MTN, the principal nucleus of the AOS in rodents, is located in the ventral portion of the midbrain tegmentum, just medial to the cerebral peduncle and the substantia nigra and lateral to the nuclear components of the ventral tegmental area. The MTN is involved in the detection of the vertical retinal slip in almost all mammals studied [27]. The MTN receives retinal input from AOT-IF and AOT-SF [26], [28]–[30]: The fibers of the AOT-IF leave the main optic tract just after passing through the optic chiasm, course caudally along the ventromedial edge of the cerebral peduncle, and terminate in the dorsal part of the MTN. On the other hand, the fibers of the AOT-SF split from the optic tract and the brachium of the SC; they then course over the surface of the cerebral peduncle, enter the ventral midbrain tegmentum, and terminate in the ventral part of the MTN [29], [30]. Despite extensive anatomical study on the connectivity between the retina and AOS nuclei, axonal pathways of the respective ON DS cell subtypes to the central structures and the functional significance of the existence of two separate pathways have not been fully elucidated.

We recently generated knock-in mice to visualize *SPIG1*-expressing cells with green fluorescent protein (GFP) [31]. SPIG1 is a secretory molecule of unknown function, which is composed of a follistatin-like domain, an extracellular calcium-binding domain, and two immunoglobulin-like domains. *SPIG1* is expressed in a unique subtype of MTN-projecting (MTN-P) ganglion cells, and their dendrites costratify with ON cholinergic amacrine strata in the inner plexiform layer, indicating that SPIG1^+^ cells are the ON DS cells [31]. The MTN-P ganglion cells are organized in two distinct mosaics comprised of SPIG1^+^ cells and SPIG1^−^ cells, suggesting that each population corresponds to one of the ON DS cell subtypes [31].

Here, we took advantage of the *SPIG1^gfp/+^* mice [31] to examine the responses of the SPIG1^+^ MTN-P cells and SPIG1^−^ MTN-P cells to the moving visual stimuli by the extracellular recordings. We found that the SPIG1^+^ MTN-P cells respond to upward motion, whereas SPIG1^−^ MTN-P cells respond to downward motion in the visual field. Retrograde labeling using the *SPIG1^gfp/+^* mice suggested that the information about upward motion and downward motion is conveyed separately to the MTN via AOT-IF and AOT-SF, respectively. In addition, dark-rearing experiments show that the preferred direction of SPIG1^+^ cells is precisely specified independently of visual experience.

## Materials and Methods

### Animals

*SPIG1^gfp/+^* mice were used for this experiment [31]. The targeted mice were backcrossed for four generations to the C57BL/6J line. Mice were reared with a 12-hr light/dark schedule. Dark-reared mice were kept in complete darkness from birth in a ventilated, light-tight chamber until the day of experiments and were inspected using infrared (IR) night-vision goggles (ATN Co, San Francisco, CA) and IR illumination. Experiments with animals were all carried out according to guidelines of the National Institute for Basic Biology (Okazaki, Japan), the RIKEN Brain Science Institute (Wako, Japan), and Senshu University (Kawasaki, Japan).

### Retrograde and anterograde labeling

Retinal ganglion cells projecting to the MTN or superior colliculus (SC) were retrogradely labeled with cholera toxin B subunit (CTB)-Alexa 555 (Invitrogen, San Diego, CA) as previously described (Yonehara et al., 2008). Dorsal part of the MTN was injected with CTB-Alexa 555 to retrogradely label the ganglion cells that project to the MTN via AOT-IF. Surface of the cerebral peduncle was injected with 1% DiI (Invitrogen) in dimethylformamide to retrogradely label the ganglion cells that project to the MTN via AOT-SF. DiI is taken into neurons from axonal tract, while CTB is taken up only from synaptic terminals. Twenty-four h (for postnatal day (P) 5 mice) or 48 h (for P10 mice) after the injection, mice were perfused with 4% paraformaldehyde. After post-fixing, retrogradely labeled retinas were removed, flat-mounted, and photographed using a fluorescent microscope (Axiphoto2; Zeiss, Jena, Germany) or a laser-scanning confocal microscope (LSM 510; Zeiss). Brains were sliced at 100 µm using a vibratome and photographed using the confocal microscope. Ganglion cell axons were anterogradely labeled with CTB-Alexa 555 as previously described [31]. Eyeballs were unilaterally injected with CTB-Alexa 555 at P5 and the central projections were analyzed at P6.

### Electrophysiological recording

At P10, the MTN (or SC) of *SPIG1^gfp/+^* mice was unilaterally injected with CTB-Alexa 555. At P12, the mice were dark adapted for 1 h prior to experiments. After cervical dislocation, the eyes were enucleated and hemisected at the ora serata under dim red light illumination. The pigment epithelium was separated from the retina and removed. Then, the retina was transferred gently to a recording chamber with the vitreal surface upward, and continuously superfused at 32°C with bicarbonate-buffered Ames solution (Sigma, St. Louis, MO) which was bubbled with 95% O_2_ and 5% CO_2_.

Extracellular recordings were obtained using a tungsten electrode (impedance, 4–7 MΩ) attached to an isolated AC differential amplifier [32] (DAM80i; World Precision Instruments, Sarasota, FL). Spike discharges were recorded from GFP^+^ ganglion cells and CTB-labeled GFP^−^ ganglion cells in the mid-peripheral region of the ventronasal retina. At first, we localized fluorescently labeled cells on fluorescence microscopy with a brief excitation (∼5 s) to minimize photobleaching. Then the identified cells and tungsten electrodes were visualized using infrared-differential interference contrast (IR-DIC) optics and displayed on a video monitor coupled to a cooled-CCD camera (DP30W; Olympus, Tokyo, Japan) mounted on the microscope. It should be noted that the tungsten electrode effectively recorded activity from cells only when placed directly atop their somata. Thus, there was no ambiguity in identification of the cell being recorded. Loose-patch recordings were also applied for GFP^+^ ganglion cells (n = 2 cells) as previously described with minor modifications [15]. Data were collected using a PC-based interface card and software.

Visual stimuli were generated by a digital micromirror device projector (PC-controlled, Custom-made model; Olympus) and presented onto a 740-µm diameter aperture of the retina. The luminance of the adaptation level was kept at 77.3 lx throughout the experiments. ON-sustained spike discharges were recorded from the fluorescently labeled ganglion cells in response to full-field diffuse light stimuli (intensity, 146.9 lx; duration, 5 s). Next, motion stimuli that traversed the receptive field in eight different directions was applied to examine direction-selective responses of the targeted ganglion cells: a drift of square-wave gratings; mean luminance, 77.3 lx; the Michelson contrast, 0.9; bright/dark duty cycle, 1∶1; each width, 300 µm on the retina; drifting speed, 10° s^−1^ in the visual angle (300 µm s^−1^ on the retina); duration, 6 s. The number of spikes obtained over a sweep of the gratings in one direction was taken as the response amplitude for the given direction. From experiments with eight directions, the preferred direction was computed as the angle of the vector sum in a polar plot of responses. Next we calculated a direction selective index (DSI) as previously described [33]. The DSI corresponds to the length of the vector sum, divided by the sum of all responses. It ranges from 0 for a cell with equal responses in all directions to 1 for a cell that responds to only one direction.

### Determination of a reference line for the retinal orientation

It was necessary to relate the preferred directions to some landmark whose position would be fairly constant among animals. In the retina of *SPIG1^gfp^* ^/+^ mice, a thick bundle of GFP^+^ axons traveling from the dorsotemporal domain of *SPIG1* expression to the optic disc is clearly seen. Two points on this bundle established the reference line to which all data for that animal would be compared. The lateral semicircular canal becomes horizontal when the nose is tilted 35° [34]. The mice were fixed at P12, and the lens and vitreous body were removed. The GFP signal in the retina was photographed using a fluorescence stereomicroscope (MZ16F; Leica Microsystems, Nussloch, Germany) with the nose tilted at 35°, and the angle of GFP^+^ bundle was calculated (n = 6 mice). This method established the angle of the reference line. The reference line was tilted 25°±1.83° (means±SD) posteriorly with respect to the true vertical line. The direction in which the bundle pointed toward the dorsotemporal domain from the optic disc was defined as 115°.

### Intracellular injection of DiI

DiI was injected as previously described [35]. In brief, retinal whole mounts taken from P12 mice were fixed in 4% paraformaldehyde in phosphate buffered saline (PBS) for 30 min. For targeting GFP^−^ MTN-P cells, the retrograde tracer CTB-Alexa 647 was injected into the MTN two days before the fixation. Sharp microelectrodes were pulled from borosilicate glass capillaries (Narishige, Tokyo, Japan). The resistance of the pipette was 30–50 MΩ, when measured with 1 M KCl. The electrode was filled with 1% DiI in 100% ethanol. The dye was injected into GFP^+^ MTN-P cells and GFP^−^ MTN-P cells with a 1–50 nA positive current for ∼1 min. After the injection, the retinas were kept in 0.4% paraformaldehyde in PBS at room temperature overnight for the efficient diffusion of dye into the fine dendrites and axons. The filled cells were photographed under a confocal microscope (LSM510). The overall morphology of the filled cells was assessed by using a 20× lens, 0.5 NA. The stratification level of the cell was analyzed using image stacks acquired with a 63× oil immersion lens, 1.4 NA, at the edges of the dendritic trees, and by measuring the intensity profiles of the DiI-labeled signal along the depth of the retina. The peaks in these profiles were used to define the stratification level of the injected cell within the inner plexiform layer (IPL). The boundaries of the IPL were defined as the peak GFP fluorescence in the ganglion cell layer (GCL) (100%), and the peak GFP fluorescence in the inner nuclear layer (INL) (0%), respectively. The direction of the dendritic arbors was computed in whole-mount micrographs. For each cell, we drew a convex polygon linking the dendritic tips, then computed the vector from the soma to the center of mass of the polygon.

### Whole-mount HRP histochemistry

The whole-mount horseradish peroxidase (HRP) histochemistry was performed as described in [36] with some modifications. At P10, mice were anesthetized with ketamine/xylazine, then given 2 µl of a 25% solution of HRP (type VI, Sigma) dissolved in 0.1 M PBS into the right eyeball using a glass micropipette. Two days later, the mice were deeply anesthetized with ketamine/xylazine and perfused with a fixative consisting of 4% paraformaldehyde and 5% dimethylsulfoxide in 0.1 M PBS. The brains were stored overnight in the same fixative at 4°C. After the cerebral cortex was removed, brainstems were incubated in 2 ml of tetramethylbenzidine buffer (TMB; ELISA POD substrate TMB kit HYPER; nacalai tesque, Kyoto, Japan) at 4°C for 30 min, and then the enzymatic reaction was initiated by adding 200 µl of H_2_O_2_ solution (ELISA POD substrate TMB kit HYPER). After the reaction at room temperature for 5 min, the brainstems were soaked in a stabilization solution (ELISA POD substrate TMB kit HYPER) at 4°C for 20 min. Immediately after the stabilization, the pathways of HRP-filled retinal fibers running on the surface of the brainstems were photographed using a stereomicroscope (MZ16F; Leica Microsystems). An image having the best-suited focus over the entire image was generated from multiple images with different focuses using Multi Focus Composition software (LEXI, Tokyo, Japan).

### OKR measurement

At P22–24, *SPIG1^gfp/+^* mice were anesthetized with ketamine/xylazine, and a metal screw was glued onto the cranial bone with dental resin (SUPER-BOND; Sun Medical, Shiga, Japan) After the surgery, the mice were dark-adapted more than 24 h until the visual stimulation. Visual stimuli were presented on a liquid crystal display monitor (Eizo, Ishikawa, Japan; 27.5 cm wide×34 cm high). The monitor was placed 14 cm from the right eye, at a 45° angle to the long body axis, covering the visual field between 0° and 90° in azimuth and −20° and 64° in elevation. Stimuli were square-wave drifting gratings (mean luminance at the cornea, 50.8 lx; the Michelson contrast, 0.9; 20° per one cycle; drifting speeds, 2° s^−1^) along the vertical or horizontal axis: It is known that the spatial vision of the mouse is tuned for lower spatial frequency than that of other tested species [37]. We used the speed of 2° s^−1^ , which is used to activate the MTN in rats [30] and guinea pigs [38]. The speed of drifting gratings was calibrated at the nearest position of the monitor from the eye. Diffuse light stimulus (intensity at the cornea; 106.9 lx) was used as a control. In each experiment, the direction and the speed of the stimulus remained constant.

The evoked eye movements were recorded by using IR video-oculographic system [39], [40]: The pupil of the right eye was illuminated by an IR light-emitting diode (Edmund optics, Tokyo, Japan). The right eye movement reflected by a hot mirror (Edmund optics) was monitored by CCD camera (XC-EI50; SONY, Tokyo, Japan). During the stimulation, the mice (P23–26) were held in a plastic cylinder with their head protruding and immobilized. The head was tilted 35° nose down so that lateral semicircular canals were positioned approximately parallel to the horizontal plane [34]. The left eye was covered with black tape. The number of eye tracking movements (ETMs) per 180 s and the gain (the angular velocity of the eye relative to the angular velocity of the stimulus) were quantitated. ETM was defined as one slow tracking movement followed by one saccade as previously described [41]. ETM assignments were made by visual inspection.

### Fos-expression mapping

The right eye of animals (P23–26) was presented with the optokinetic visual stimulus for 1 h. Essentially no adaptation was observed in OKR during the period of stimulation (data not shown). After the stimulation period, the mice were deeply anesthetized with an overdose of ketamine/xylazine and perfused with 4% paraformaldehyde. Brains were removed and post-fixed overnight at 4°C. The midbrain containing the MTN was cut in the coronal plane (50 µm) using a vibratome. Consecutive sections containing the MTN were processed for Fos immunoreactivity. The sections were rinsed in PBS containing 0.1% Triton-X for 10 min and blocked with 10% horse serum/PBS containing 0.1% Triton-X for 1 h at room temperature. They were then incubated with rabbit anti-Fos (dilution 1∶1000, sc-52; Santa Cruz Biotechnology, Santa Cruz, CA) in 10% horse serum/PBS containing 0.1% Triton-X overnight at 4°C. The sections were next washed with PBS at room temperature, and incubated with Alexa 546 goat anti-rabbit IgG (dilution 1∶400, A11035; Invitrogen) for 2 h at room temperature. Finally, they were washed with PBS, mounted in Permafluor (Beckman Coulter, Fullerton, CA), and cover-slipped.

Images were acquired using a confocal microscope (LSM 510) with a 10× objective (N.A. 0.3; Zeiss) (optical section; 50 µm). Boundaries of the MTN were identified from GFP signals of retinal axons innervating the MTN. The MTN was subdivided into the dorsal division (MTNd) and the ventral division (MTNv) according to histological criteria described before [42]. The MTNd was further subdivided into upper and lower halves. The MTNv was further subdivided into dorsal and ventrolateral parts. Fos-positive cells in each subregion on the contralateral side were counted in consecutive sections covering the entire nucleus.

## Results

### MTN-P ganglion cells form two distinct regular mosaics

**Figure 1:**
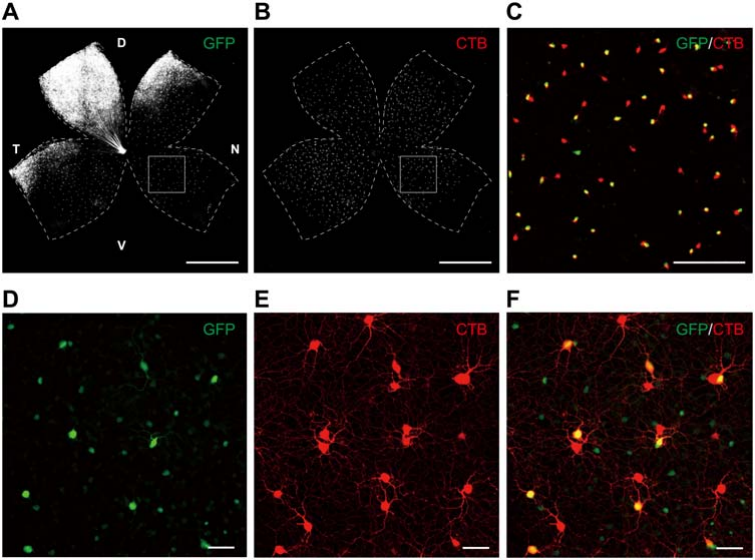
SPIG1 marks half the population of the MTN-projecting cells in the mouse retina. A, SPIG1^+^ cells labeled by GFP (green) in the flat-mount retina of *SPIG1^gfp/+^* mice at P6. The retina is apparently subdivided into two domains with *SPIG1* expression, the dorsotemporal domain and pan-ventronasal domain. The picture is shown in black and white for clarity. B, MTN-projecting (MTN-P) cells were retrogradely labeled with cholera toxin B subunit (CTB)-Alexa 555. MTN-P cells are actually labeled in red. C, The enlargement of the boxed region in A and B. MTN-P cells are subdivided into two populations, GFP^+^ (yellow: due to both red and green colored) and GFP^−^ (red) cells. D, GFP^+^ cells in the ventronasal region of flat-mount retina at P12. By P10, presumptive amacrine cell subsets with a small somatic diameter also begin to express GFP. E, MTN-P cells were retrogradely labeled with CTB-Alexa 555 (red). It is recognizable that dendrites of the MTN-P cells completely tile the retina. F, A merged picture of D and E. It is apparent that one of the paired MTN-P cells is always GFP^+^. D, dorsal; N, nasal; T, temporal; V, ventral. Scale bars: A, B, 1 mm; C, 200 µm; D–F, 50 µm.

We examined the GFP expression in the whole-mount retina of *SPIG1^gfp/+^* mice at P6 (Figure 1A–C) and P12 (Figure 1D–F). When we injected a tracer into the MTN, the principal nucleus of the AOS [25], [27], half of the labeled cells were overlapped with GFP^+^ cells; over 95% of GFP^+^ ganglion cells in the pan-ventronasal domain were retrogradely labeled at P6 and P12 (Table 1). The MTN-P ganglion cells formed two distinct but interdependent regular mosaics in the P6 retina (Figure 1A–C; see GFP-expression plus (yellow) and minus (red) in Figure 1C), and they are already detected as early as P1 [31]. These features indicated that the MTN-P cells comprise two functionally distinct subtypes of ON DS cells and that SPIG1^+^ cells correspond to one of the subtypes. When analyzed at P12, GFP was also expressed in presumptive amacrine cell subsets located in the GCL and INL, in addition to the MTN-P cells (Figure 1D–F, small GFP^+^ cells; see also [31]). Because the expression level of GFP in amacrine cells was weak and the diameter of their somata was smaller (<10 µm) compared with that of MTN-P cells (∼19 µm), it was easy to distinguish them at P12.

**Table 1:** Numbers of retrogradely labeled RGCs in the contralateral retina.

Injection site	pan-VN domain			DT domain
	Labeled GFP^+^	Labeled GFP^−^	Unlabeled GFP^+^	Labeled GFP^+^
AOT-SF (n = 3)	5.3±2.3	207.0±28.1	281.3±21.4	44.3±13.0
MTN (n = 3)	279.0±14.5	324.0±7.6	10.0±2.6	226.3±20.4

Data are expressed as mean±SEM. Pan-VN, pan-ventronasal; DT, dorsotemporal.

### SPIG1 expression marks ON DS cells that respond to upward motion

**Figure 2:**
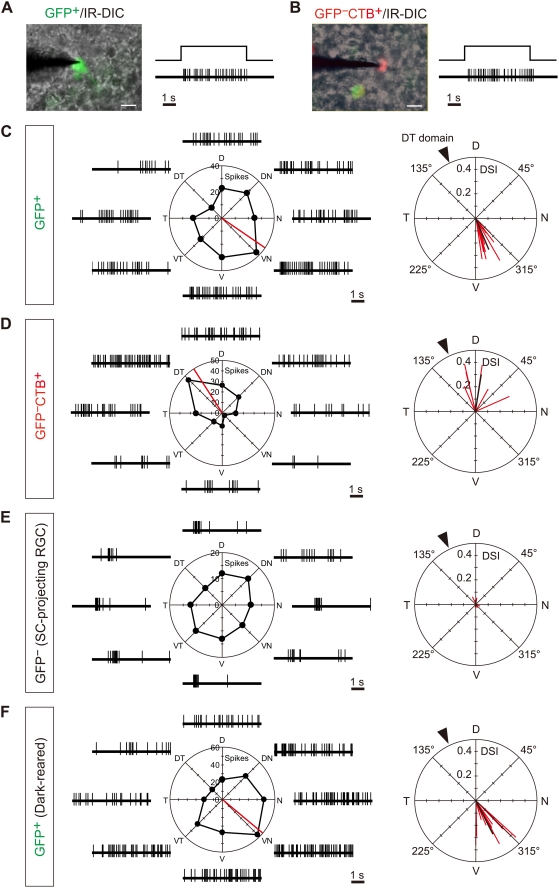
SPIG1 marks the upward-preferring subtype of ON DS cells. A, B, Left, the tip of a tungsten electrode was attached onto a GFP^+^ MTN-P cell (A) or GFP^−^ MTN-P cell (B) to record spike discharges from the cell. Cells were identified by using IR-DIC optics. Right, responses of a GFP^+^ MTN-P cell (A) and GFP^−^ MTN-P cell (B) to a light stimulus for 5 s. Both showed sustained responses during the light stimulation. C–F, Left panels, representative spike trains and their polar plots in response to eight different directions of the drifting square-wave gratings for 6 s. Spikes were recorded from a GFP^+^ MTN-P cell (C), GFP^−^ MTN-P cell (D), SC-projecting cell (E), and GFP^+^ MTN-P cell in dark-reared mice (F), respectively. The red line indicates the preferred direction of the cells. Right panels, preferred direction and direction selectivity index (DSI) of individual cells are represented by the angle and length of the red line, respectively. The black line indicates the average of the preferred direction and DSI. The DSI indicates the degree of asymmetry in the polar plot of responses to the drifting gratings (see Materials and Methods). The arrowhead indicates the direction of the dorsotemporal domain. Scale bars: A, B, 20 µm.

To verify that GFP^+^ ganglion cells projecting to the MTN correspond to ON DS cells, we directly recorded spike discharges from these cells in the pan-ventronasal domain in whole-mount retinal preparations of *SPIG1^gfp/+^* mice (Figure 2). Because the expression of *SPIG1* in retinal ganglion cells begins to decrease after eye-opening [31], experiments were performed on retinas at P12, a day just before eye-opening. MTN-P cells were labeled with a retrograde tracer from the MTN in advance to identify GFP^−^ MTN-P cells. Of note is that both GFP^+^ MTN-P cells and GFP^−^ MTN-P cells showed ON-sustained responses to a full-field light stimulus (Figure 2A, B), which is a characteristic property of ON DS cells [15].

To explore the responses of the retinal ganglion cells to motion, we next moved square-wave gratings upon the retina in eight different directions. Importantly, all GFP^+^ MTN-P cells examined (n = 10 cells) had the same preferred direction; they responded strongly to the motion in the dorsal to ventral direction (Figure 2C; preferred direction, 292.7°±13.3°, mean±SD; direction selective index (DSI), 0.266±0.075, mean±SD). Similar results were obtained by loose-patch recording (n = 2, data not shown). Because an image is reversed by the lens, these cells detect upward motion in the visual field. In contrast, all GFP^−^ MTN-P cells (n = 6) responded strongly to the motion in the ventral to dorsal direction (Figure 2D; preferred direction, 81.1°±33.9°; DSI, 0.307±0.077). As a control, SC-projecting (SC-P) ganglion cells were also identified by retrograde labeling, and examined for the direction selectivity. Among the SC-P cells that showed ON-sustained responses (n = 7), none showed apparent direction selectivity expectedly (Figure 2E; DSI, 0.038±0.013). Thus, MTN-P cells comprise two physiological subtypes of ON DS cells, namely upward-preferring and downward-preferring subtypes, and SPIG1 marks exclusively the upward-preferring subtype.

### Visual experience is not a prerequisite for the specification of preferred direction

Previous studies showed that visual stimuli through closed eyelids have striking effects on the development of the visual circuit [43]. We therefore examined the requirement of visual stimuli by light for the development of direction selectivity of GFP^+^ MTN-P cells. Mouse pups were reared in the dark from birth, and the direction selectivity of GFP^+^ ganglion cells was examined at P12 (Figure 2F). GFP^+^ ganglion cells in dark-reared mice showed almost the identical preferred direction and DSI (preferred direction, 296.9°±17.2°; DSI, 0.306±0.094; n = 10) as those in normal (light-reared) mice (p = 0.542 for preferred direction; p = 0.305 for DSI, Student's *t*-test), indicating that visual experience is not a prerequisite for the development of direction selectivity and the specification of preferred direction in SPIG1^+^ ON DS cells.

### ON DS cell subtypes are morphologically indistinguishable

**Figure 3:**
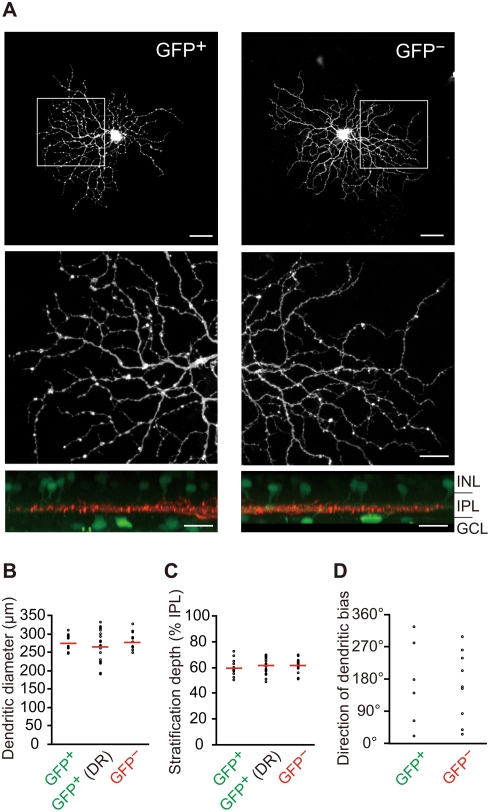
Morphological features do not indicate the preferred direction. A, Examples of a GFP^+^ MTN-P cell (left column) and a GFP^−^ MTN-P cell (right column) injected with DiI intracellularly. A bird's eye view (xy plane; top), enlargement of the boxed region (middle), and side view (xz plane; bottom) are shown for each cell at P12. The boundary of the IPL is apparent from the array of the somata (green) of GFP^+^ cells located in the GCL (GFP^+^ amacrine cells and ganglion cells) and INL (GFP^+^ amacrine cells) in the xz plane. Scale bars: top, 50 µm; middle and bottom, 20 µm. B, The dendritic field diameter. DR, dark-reared mice. Circles, data for individual cells; lines, means. C, The dendrite stratification depth. The stratification depth was defined as 0–100% from the INL side to the GCL side of the IPL. D, Direction of dendritic bias. The angle represents the difference between the nasal direction and the direction of dendritic bias. For the analysis of dendritic bias, only cells in the mid-peripheral region of the ventronasal quadrant in the retina were analyzed (n = 6 for GFP^+^ MTN-P cells, n = 11 for GFP^−^ MTN-P cells).

To estimate possible morphological bias that predicts preferred direction in the retina, we examined the dendritic morphology of individual GFP^+^ MTN-P cells (n = 16 cells) and GFP^−^ MTN-P cells (n = 15) by single-cell injection with DiI at P12 (Figure 3A). We could not find any significant differences in morphological features between these two subtypes in the following three criteria: dendritic field diameter (Figure 3B; p = 0.773, Student's *t*-test), stratification depth within the IPL (Figure 3C; p = 0.269, Student's *t*-test), and direction of dendritic bias (Figure 3D; p = 0.841, Mann-Whitney *U* test), in consistent with a previous report [44]. Furthermore, dark-rearing did not exert significant effects on the dendritic field diameter (p = 0.332, Student's *t*-test) and stratification depth (p = 0.308, Student's *t*-test) of GFP^+^ MTN-P cells (Figure 3B, C; n = 22).

### MTN receives retinal input via AOT-IF and AOT-SF

**Figure 4:**
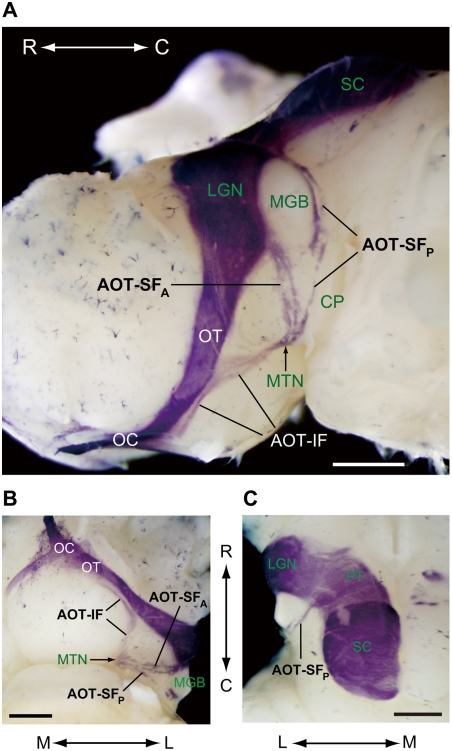
MTN receives retinal inputs from AOT-IF and AOT-SF. Retinal central pathways were visualized by the whole-mount HRP histochemistry on a mouse brain after the injection of HRP into the right eyeball. A, A lateral view. B, A ventral view. C, A dorsal view. The main pathway toward the lateral geniculate nucleus (LGN) and the SC, and the accessory optic pathways consisting of the AOT-IF and AOT-SF toward the MTN are visible as HRP-positive. The AOT-SF is further segregated into the anterior fibers of the AOT-SF (AOT-SF_A_) and the posterior fibers of the AOT-SF (AOT-SF_P_). CP, cerebral peduncle; MGB, medial geniculate body; OC, optic chiasm; OT, optic tract; PT, pretectal area; R, rostral; C, caudal; L, lateral; M, medial. Scale bars: 1 mm.

To visualize the three-dimensional fiber pathways from the retina to the MTN, we performed whole-mount HRP histochemistry (Figure 4A–C). HRP was injected into the right eyeball at P10, and HRP-filled fibers were visualized by TMB method at P12. The overall features of the central pathways of AOS were well consistent with that in a previous report in which HRP-filled fibers were analyzed in brain sections in the mouse [29]. The fibers of the AOT-IF left the main optic tract at the level of the midhypothalamus, coursed caudally along the ventromedial edge of the cerebral pedundle, and terminated within the MTN (Figure 4A, B). The AOT-SF was composed of two groups of accessory optic axons: the anterior and posterior fibers of the AOT-SF. The anterior fibers derived from optic tract mainly at the level just beneath the ventral lateral geniculate nucleus (Figure 4A; AOT-SF_A_). The posterior fibers emerged from the brachium of the SC and ran ventrolaterally around the caudal portion of the medial geniculate body (Figure 4A, C; AOT-SF_P_). Then the anterior and posterior fibers coursed over the surface of the cerebral peduncle and terminated within the MTN (Figure 4A, B).

### GFP^+^ ganglion cells and GFP^−^ ganglion cells project to the MTN via different optic pathways

**Figure 5:**
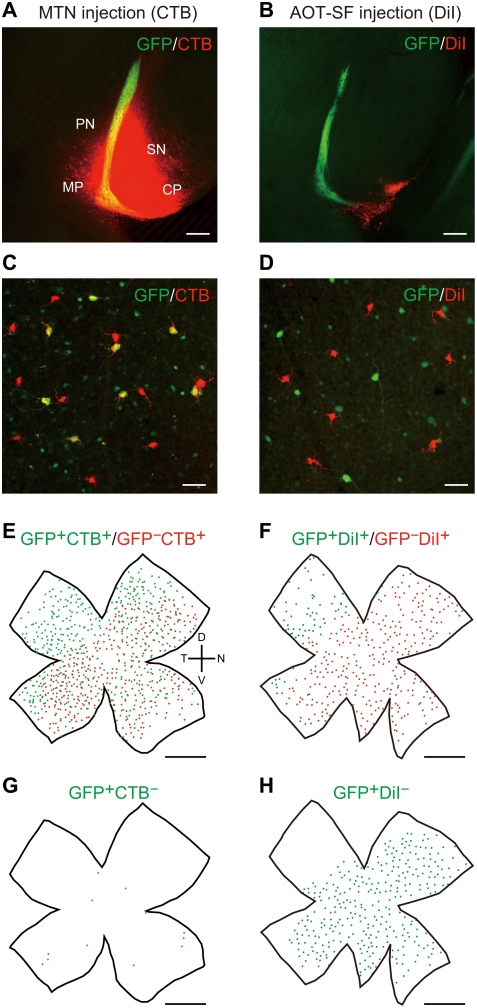
AOT-SF specifically conveys the information about downward image motion. CTB-Alexa 555 or DiI was injected into the MTN (left column) or AOT-SF (right column), respectively, to retrogradely label ganglion cells. A,B, The injection sites in coronal slices at P12. C, D, Retrogradly labeled ganglion cells in the pan-ventronasal domain of the contralateral retina. E, F, Representative reconstructions of the distribution of the retrogradely labeled ganglion cells in a flat-mount retina at P12. Retrogradely labeled cells in the pan-ventronasal domain are subdivided into two populations; GFP^+^CTB^+^ cells (green dots) and GFP^−^CTB^+^ cells (red dots) for MTN injection (E), and GFP^+^DiI^+^ cells (green dots) and GFP^−^DiI^+^ cells (red dots) for AOT-SF injection (F). Note that in the dorsotemporal domain all retrogradely labeled cells are GFP^+^. G, H, The distribution of GFP^+^ ganglion cells that were not labeled retrogradely in the pan-ventronasal domain (green dots). A small number of GFP^+^ cells which probably project to ipsilateral MTN are not labeled with CTB (G). GFP^+^DiI^−^ cells (H) correspond to blue cells in Figure 7A. The cells in the dorsotemporal domain are not shown, because they are too crowded. CP, cerebral peduncle; MP, mammillary peduncle; SN, substantia nigra; PN, paranigral nucleus. Scale bars: A, B, 200 µm; C, D, 50 µm; E–H, 1 mm.

Because the MTN receives retinal input via AOT-SF and AOT-IF, we tested the possibility that information about upward motion and downward motion are separately conveyed to the MTN via these two pathways. We unilaterally injected CTB-Alexa 555 or DiI into the MTN (n = 3 mice; Figure 5A) or AOT-SF (n = 3 mice; Figure 5B), respectively, at P10, and examined the contralateral retina at P12 (Figure 5C, D). Under both conditions, retrogradely labeled ganglion cells were distributed throughout the retina (Figure 5E, F). Approximately half (∼46%) of the CTB-labeled cells in the pan-ventronasal domain were GFP-positive after MTN injection (Figure 5E, G; Table 1; 279/603). In contrast, almost all (∼98%) of the DiI-labeled cells were GFP-negative when AOT-SF was labeled with DiI (Figure 5F, H; Table 1; 207/212). In the dorsotemporal domain, all labeled cells were GFP-positive under both conditions (Figure 5E, F). Quantitative analysis of retrogradely labeled ganglion cells in the pan-ventronasal domain indicated that AOT-SF corresponds to axons of GFP^−^ MTN-P cells (ANOVA interaction between injection site and GFP expression: F_1,4_ = 22.1; p = 0.0093; Table 1).

Retinal fibers via AOT-IF was supposed to terminate in the MTNd [30]. To label these fibers, we injected a tracer into the whole of the MTN (n = 3 mice; Figure S1, A, upper panel) or the MTNd (n = 2; Figure S1, A, lower panel) at P5, and characterized retrogradely labeled ganglion cells in the contralateral retina at P6 (Figure S1, B). Approximately 350 and 80 GFP^−^ MTN-P cells were retrogradely labeled in the pan-ventronasal domain at P6 after injection into the whole of the MTN and MTNd, respectively (red cells in Figure S1, C). These results suggest that most of the MTN-P ganglion cells that course through AOT-IF and innervate MTNd are GFP^+^ ganglion cells, and that most of the GFP^−^ ganglion cells selectively innervate the MTNv.

Next we labeled all ganglion cell axons by injecting the eyeball with an anterograde tracer and examined the MTN in coronal sections (n = 3 mice; Figure S2, A) and parasagittal sections (n = 3 mice; Figure S2, B). The AOT-IF was observed to enter the MTN and terminate in the MTNd in parasagittal section (Figure S2, B), whereas the AOT-SF was observed to enter the MTNv where it terminates in both coronal and parasagittal sections (Figure S2, A, B). We found again that axons belonging to the AOT-IF and terminating in the MTNd are predominantly positive for GFP (Figure S2, B, lower panels), whereas axons belonging to the AOT-SF are almost negative for GFP (Figure S2, A, B, upper panels). Retrograde and anterograde labeling thus demonstrated that axons of GFP^+^ cells innervate the MTNd via AOT-IF, whereas axons of GFP^−^ cells innervate the MTNv via AOT-SF, if not exclusively.

### MTN neurons receive information about upward and downward visual motion

**Figure 6:**
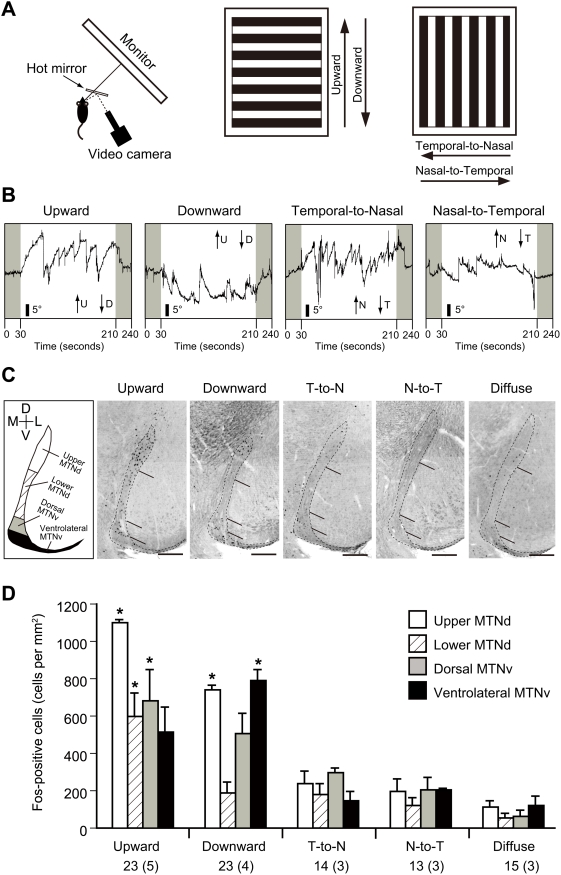
MTN is activated by the vertical image motion. A, Visual stimuli used to induce the OKR. Stimuli were drifts of square-wave gratings moving in four different directions (middle and right panels). Movements of the right eye were recorded during visual stimulation of the right eye (left panel). B, Vertical OKR and horizontal OKR evoked by monocular stimulation. Uniform gray light was presented during the first and last 30 seconds. C, Motion-induced activation of the MTN estimated from Fos expression. The MTN was subdivided into four subregions: upper and lower halves of the MTNd, and dorsal and ventrolateral parts of the MTNv. Diffuse light without motion was used as a control. Coronal sections of the MTN were immunostained with anti-Fos antibodies to detect the expression of the immediate-early gene Fos. Only nuclear signals within the MTN (enclosed by dashed lines) were counted. Note that signals dorsomedial to the MTN are mostly derived from fibers and apparently non-specific signals. T, temporal; N, nasal. Scale bars: 200 µm. D, Quantification of Fos^+^ cells in the four subregions of the MTN. Numbers of the sections and animals used are indicated below. Error bars indicate SE. *p<0.05, Turkey-Kramer test.

We next examined that the information about vertical visual motion that can induce vertical OKR is indeed conveyed to the MTN. To record the vertical OKR and horizontal OKR, we combined IR video-oculographic system [39], [40] and a computer display system for visual stimulation (Figure 6A). Experiments were performed at P23–26 (n = 7 mice), a few days after the OKR is first observed in mice [45]. Upward and downward motion of square-wave gratings at 2° s^−1^ induced vertical OKR, which is characterized by the repeat of one slow eye tracking movement (ETM) followed by one resetting saccade of the eyeball (Figure 6B). Similarly, horizontal OKR was induced by temporal-to-nasal and nasal-to-temporal motion of the stimuli (Figure 6B). We noticed that the dynamics of eye movements evoked by upward and downward optokinetic stimulation are different: Unlike OKR to upward motion, OKR to downward motion was interspersed with fewer fast resetting saccadic eye movements (ETMs/180 s, 8.43±1.90 for upward motion, 1.29±1.60 for downward motion, mean±SD; p<0.0001, Student's *t*-test), but the gain was the same (gain, 0.394±0.057 for upward motion, 0.387±0.106 for downward motion, mean±SD; p = 0.893, Student's *t*-test). Moreover, vertical eye deviations greater than 10° were often maintained without resetting in OKR to the downward motion (Figure 6B).

Next we examined the effects of these optokinetic stimuli on the activity of the MTN by the expression of Fos, an immediate-early gene product, at P23–26 (Figure 6C, D). As expected from our electrophysiological experiments, upward (n = 5 mice) and downward (n = 4) motion of square-wave gratings at 2° s^−1^ strongly activated Fos expression in the contralateral MTN, as compared with diffuse light without motion given as a control (n = 3). However, temporal-to-nasal (n = 3) and nasal-to-temporal (n = 3) motion did not activate it significantly (Figure 6C, D). The specificity of the Fos expression following vertical visual motion suggests that the MTN relays visual signals for the control of vertical OKR in mice. In the ipsilateral MTN, Fos expression was not induced by motion in any directions (data not shown). In addition, interestingly, we found that upward motion activated the MTN stronger than downward motion on the whole (Figure 6C, D): Upward motion predominantly activated the upper half of the dorsal division of the MTN (MTNd) also with the dorsal part of the ventral division of the MTN (MTNv). In contrast, downward motion predominantly activated the upper half of the MTNd and the ventrolateral part of the MTNv.

## Discussion

Previously, we reported that the MTN-projecting cells in the pan-ventronasal domain of the retina form two distinct regular mosaics overlaying with equal density [31]. Here we show that the each mosaic population corresponds to a physiological subtype of ON DS cells with distinct preferred direction, upward or downward. It has been shown that ON-OFF DS cells are organized in four overlaid tilings, each tiling consisting of like-type cells that respond to a particular direction and that closely spaced pairs of the DS cell somata comprise different subtypes of DS cells [46], [47]. Therefore, it may be possible to say that overlaid mosaics of DS cells with different preferred directions are a general feature of the organization for information outflow from the retina to the brain. However, when compared to ON-OFF DS cells, the dendritic tiling of ON DS cells is less precise. Retinal coverage factor for each subtype of MTN-P cells at P12 is on the order of 3.0, when it was calculated from the dendritic field area of DiI-filled cells (Figure 3B) and the density of these cells in the pan-ventronasal domain. On the other hand, estimated coverage factor for each functional subtype of ON-OFF DS cells is reportedly on the order of 1.0 [48]–[50]. The marked difference in the coverage factor between two DSGC types may reflect differences in the structure of retinal circuitry underlying the direction selectivity.

In contrast to the direction selectivity in the visual cortex [51], the direction selectivity in the retina has been thought to be established without visual stimulus [52], [53]. Recent study using multielectrode array also showed the presence of four subtypes of ON-OFF DS cells in mice that have been reared in darkness and in mice that lack the β2 subunit of the nicotinic acetylcholine receptor [54]. However, it is not clear whether preferred direction of each DS cell is completely specified independent of retinal activity. Our results indicate that the preferred direction for SPIG1^+^ ganglion cells, one of the ON type DS cells, is specified as upward-preferring independently of visual experience (Figure 2F). The strength of direction selectivity of SPIG1^+^ subtype was not affected by the dark-rearing before eye-opening (Figure 2F), along with the dendritic morphology (Figure 3B, C). Very recently, similar results were reported for ON-OFF DS cells in rabbits [55]. These results altogether support a view that the establishment of direction selectivity is not dependent on visual experience. This view is consistent with the previous findings in a different species in that eye rotation during development has no effect on the direction-selectivity of neurons in the nucleus of the optic tract in wallaby [56]. Moreover, visual experience is not essential for the development of OKR in rabbits [57].

We have already provided the evidence that the physiological subtypes of ON DS cells are molecularly distinguishable from early in development: SPIG1 marks a small population of postmitotic ganglion cells as early as embryonic day (E)15.5 [31]. The MTN begins to be innervated by GFP^+^ axons at E17.5. SPIG1^+^ and SPIG1^−^ ganglion cells projecting to the MTN form distinct regular mosaics as early as P1. The two subtypes of ON DS cells are inclined to reside as pairs in the retina, suggesting an interdependency in the cellular differentiation during development. The existence of segregated central projection of two ON DS cell subtypes suggests that each subtype express distinct guidance molecules at an embryonic stage. Therefore, it is likely that SPIG1^+^ and SPIG1^−^ MTN-P cells are genetically destined for each subtype that responds to upward and downward motion, respectively, already at the onset of central projection. Such an early divergence of subtypes might be controlled by morphogen signals and transcription factors which are asymmetrically expressed in the developing retina [58]–[62]. Intriguingly, the preferred direction of GFP^+^ ganglion cells, namely SPIG1^+^ ganglion cells, in the retina coincides with the direction in which GFP^+^ axon bundles from the dorsotemoral domain pointed toward the optic disc (Figure 1A; tilted about 25° posteriorly from the dorso-ventral axis). It is also tempting to speculate that molecular gradients along retinal axes play a role in the establishment of spatially asymmetric connections between DS cells and starburst amacrine cells.

We here report a first demonstration of vertical OKR in rodents, although horizontal OKR in rodents has been analyzed by many research groups [34], [37], [45], [63]–[68]. We found a directional asymmetry in vertical OKR; downward motion induced a smaller number of ETMs compared with the upward motion in our stimulation paradigm (Figure 6B). Directional asymmetries of vertical OKR have been studied in other species such as humans [69], monkeys [70], cats [71], rabbits [72], and chickens [73]. However, the gains of vertical OKR to upward and downward motion were similar and both of them were 0.39, suggesting that the asymmetry in the vertical OKR is likely related to saccadic mechanisms. Our results also indicate that retinal slip upon the visual motion stimulus constantly occurs during the stimulation period. This observation suggests that the retina actually keeps on receiving the visual stimulus moving only in one direction (upward or downward) during the stimulation period for Fos expression analysis. The speed of image motion in the retina is estimated to be 1.2°–2°/s. Because the speed of resetting saccades is fast, ON DS cells should not be activated during the saccades.

The direction-selective responses in the mammalian AOS nuclei show species differences, which may reflect the differences in their lifestyles. In rabbits, the majority of neurons in the MTN prefer upward motion [19], whereas in the cats, they prefer downward motion [74]. By analyzing the Fos expression, we first examined the direction selectivity of MTN neurons in the mice. We showed that equal number of upward-preferring cells and downward-preferring cells project to the MTN, suggesting that the direction selectivity of the MTN neurons are essentially formed by MTN-P cells. Temporal-to-nasal motion did not induce the Fos expression in the MTN (Figure 6C, D), indicating that the ON DS cell subtype which prefers temporal-to-nasal direction projects to the DTN, because neurons in this nucleus prefer temporal-to-nasal direction as reported in other species [16]. Actually, we found that the DTN is activated by temporal-to-nasal motion in mice (data not shown). It is of note that the LTN, which responds to the vertical visual motion as reported in other species [16], is not observed consistently in mice [29]. We found that Fos expression is induced in the MTN neurons at P23–26 but not at P14 (data not shown), although MTN-P cells show direction-selective responses at P12, just before the eye-opening (Figure 2). Considering that OKR is first observed at three weeks of age in mice [45], it is possible that ability of MTN neurons to express Fos protein is tightly correlated with the maturity of their function.

**Figure 7:**
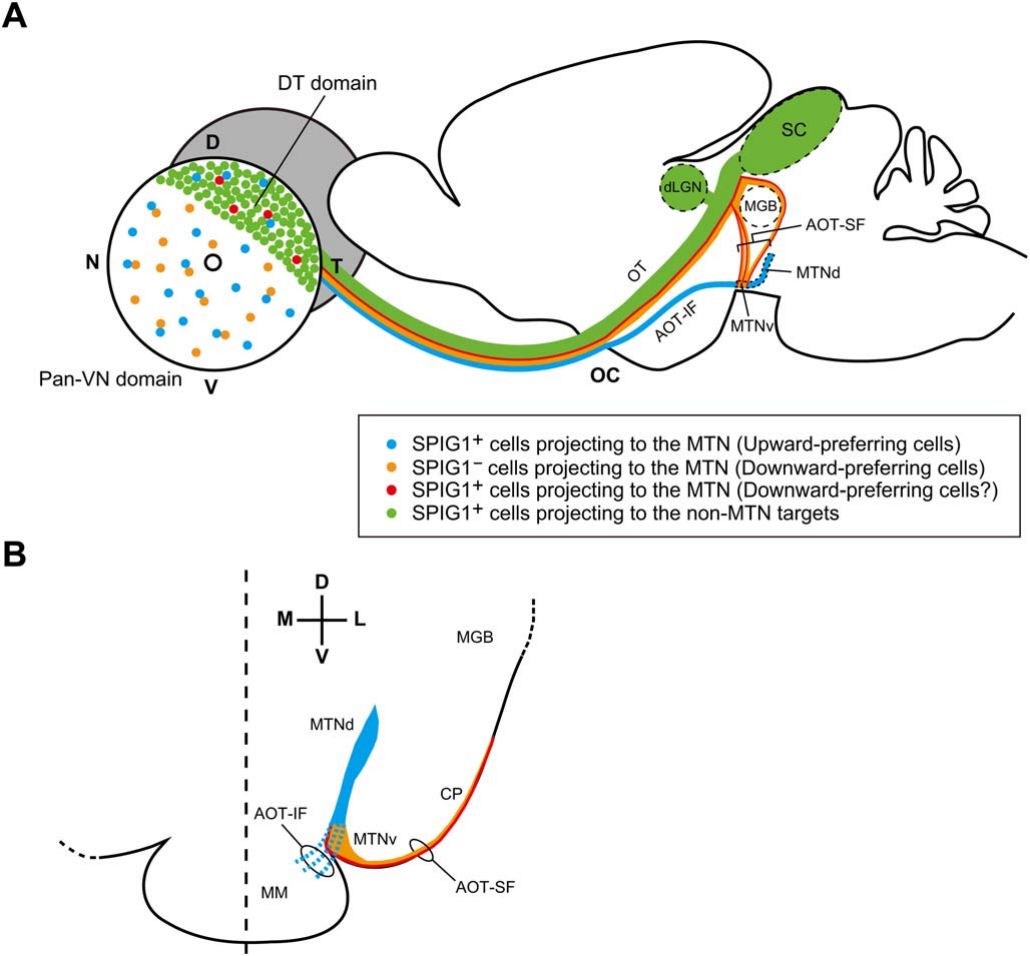
Schematic representation of axonal connectivity between the retina and the contralateral MTN of the AOS. Information of upward and downward visual motion is conveyed to the MTN by distinct neuronal pathways. This represents our findings together with those in the previous studies on the retinal projection to the MTN in mice and rats [29], [30]. A, Upward-preferring subtype of ON DS cells (SPIG1^+^; blue) predominantly projects to the MTNd via AOT-IF, whereas downward-preferring subtype of ON DS cells (SPIG1^−^; orange) projects to the MTNv via AOT-SF. MTN-projecting cells in the dorsotemporal domain are all SPIG1^+^, but subdivided into two subtypes: one that courses through AOT-IF (blue) and the other AOT-SF (red). Most of the SPIG1^+^ ganglion cells in the dorsotemporal domain (green) project to the dorsal lateral geniculate nucleus (dLGN) and SC via optic tract (OT). The fibers of the AOT-SF split from the OT and the brachium of the SC, then they course ventrally over the surface of the cerebral peduncle (CP) and finally terminate in the MTNv. On the other hand, the fibers of the AOT-IF leave the OT just after passing through the optic chiasm (OC), then course caudally, and terminate in the MTNd. MGB, medial geniculate body. B, View in the coronal section at the level of the MTN. The superficially lying AOT-SF enters the MTNv and terminates there. AOT-IF, which is a dispersed group of fibers, enters the MTN from the rostral side and terminates in the MTNd. MM, medial mammillary nucleus.

The three-dimensional organization of AOT-IF and AOT-SF in the mouse was more similar to that in the hamster than that in either the rat or guinea pig [36], as previously described [29]. By combining genetic labeling, retrograde labeling, and targeted electrophysiological recordings, here we provide direct evidence that axons of downward-preferring subtype of ON DS cells course through the AOT-SF and largely terminate in the MTNv (Figure 7; Figure 5; Figure S2), whereas axons of upward-preferring subtype of ON DS cells course through the AOT-IF and terminate in the MTNd (Figure 7; Figure S1; Figure S2). This was proposed previously [30] based on the following several observations in rats: First, neurons in the dorsomedial portion of the MTN are predominantly stimulated by upward movements and those in the ventrolateral portion of the MTN by downward movements. Second, a correlation was found between the segregation of direction-selective units and the areas in the MTN to which the retinal fibers from the two pathways distribute. In the present study, we also show that neurons in the MTNd are best activated by the upward motion and neurons in the ventrolateral part of the MTN are best activated by downward motion (Figure 6), in mild agreement with the observation in rats [30], [75]. The lateral part of the MTNv may correspond to the interstitial nucleus in the posterior bundle of the superior fasciculus, which has been functionally characterized in the guinea pig [76]. In the guinea pig, this nucleus was activated by the vertical visual motion, although it was activated equally by the upward and downward motion, when estimated by the uptake of ^14^C-2-deoxyglucose [76]. The interstitial nucleus in the posterior bundle of the superior fasciculus is a sparse assemblage of neurons scattered throughout the course of the AOT-SF. In the present study, ventrolateral part of the MTNv, which is an entry zone for the fibers of AOT-SF into the MTN, was preferentially activated by the downward motion as shown by the Fos expression (Figure 6). This also strongly supports our conclusion that the AOT-SF specifically conveys the information about downward visual motion. It is still an open question whether segregated projection and discrete clusters of axon terminals are also seen for ON-OFF DGSC subtypes.

Based on the number of retrogradely labeled SPIG1^−^ MTN-P cells in the pan-ventronasal domain and SPIG1^+^ MTN-P cells in the dorsotemporal domain after MTN and AOT-SF injection (Table 1), we estimated that approximately 70 cells and 160 cells in the dorsotemporal domain course through AOT-SF and AOT-IF, respectively. This observation implies that the MTN-P cells in the dorsotemporal domain are also subdivided into two subtypes of ON DS cells, although not evenly. This view is supported by the finding that weak GFP signals are observed in the AOT-SF (Figure S2, A). By our MTN injection with CTB, approximately 10 GFP^+^ ganglion cells were not labeled in the pan-ventronasal domain (Figure 5G, Table 1; please note that one GFP^+^CTB^−^ cell is present in Figure 1C). This is attributable to the finding that some of the unlabeled cells projects to the ipsilateral MTN [31]. Results of the MTNd injection may suggest that one-fifth of the SPIG1^−^ MTN-P cells that course through the AOT-SF terminates in the MTNd (Figure S1, C, compare the number of GFP^−^ cells in the upper and lower drawings). This view is consistent with our observation that neurons in the upper half of the MTNd were significantly activated by the downward motion (see Figure 6D, Downward, Upper MTNd). Alternatively, it is possible that the CTB injected into the MTNd was actually diffused into the MTNv, or that the segregation of axon terminals in the MTN was not completed at P6, a few days before ganglion cells make synapses with bipolar cells [77]. We also found that dorsal part of the MTNv was activated by upward motion (see Figure 6C, D; Upward, Dorsal MTNv). Because AOT fibers extending to the MTNd emit many terminal collaterals within the MTN [78], it is assumable that axon terminals of upward-preferring subtype make synapses with neurons located at MTNv as well as at MTNd.

The MTN has several important connections to midbrain and brainstem structures to be involved in vertical OKR [79]. Notably, MTN neurons send visual signals to the dorsal cap of inferior olive largely via the visual tegmental relay zone, which integrates upward and downward signals to produce bipartite receptive fields that respond best to rotation about an axis related to the vestibular semicircular canals [80]. However, the structure of neural circuits that creates the bipartite receptive fields in the visual tegmental relay zone remains unknown.

Together with recent reports [33], [81], our study demonstrated that the genetic labeling of a specific subtype of retinal ganglion cell is a useful method for relating the mosaic spacing, dendritic morphology, and central projection of the subtype to its electrophysiology. Our characterization of ON DS cells may provide insights into how information about the direction of image motion is extracted in the retina, conveyed to the brain, and utilized for the production of the OKR in vertebrates. Especially, further studies will pave the way for our understanding of the molecular and cellular mechanism underlying the formation of the direction-selective retinal circuitry.

## Supporting Information

Figure S1 Projection sites of upward- and downward-preferring subtypes are segregated in the MTN. A, Examples of injection of the retrograde tracer CTB-Alexa 555. The whole of the MTN (upper) or the MTNd (lower) received an injection. B, Retrogradely labeled retinal ganglion cells in the pan-ventronasal domain of contralateral retinas at P6. C, Representative drawings of retrogradely labeled cells in the contralateral retinas at P6. CTB^+^ cells (MTN-P cells) are subdivided into two populations; GFP^+^CTB^+^ cells (green dots) and GFP^−^CTB^+^ cells (red dots). The number of GFP^+^CTB^+^ cells and GFP^−^CTB^+^ cells in the pan-ventronasal domain was: 1143 and 1250 (summed over 3 retinas), respectively, when the whole area of the MTN was injected; 708 and 170 (summed over 2 retinas), respectively, when the MTNd region was injected. This result indicates that projection sites of GFP^+^CTB^+^ cells and GFP^−^CTB^+^ cells are significantly segregated along the dorsoventral axis of the MTN (χ^2^ = 282.6; p<0.0001). Scale bars: A, 200 µm; B, 100 {lwoer case mu}m; C, 1 mm.
(7.18 MB TIF)

Figure S2 MTN receives retinal axons from AOT-IF and AOT-SF. All retinal ganglion cell axons from an eye had been labeled anterogradely by CTB-Alexa 555 one day before the mice were killed (n = 3 mice for each coronal and parasagittal section). A, Coronal section at P6. The superficially lying AOT-SF (black arrowhead) is entering the MTNv (white arrowhead) to terminate in this region. AOT-SF and MTNv show weaker GFP signals than the MTNd (arrow). B, Parasagittal section at P6. Upper panels, the AOT-IF (double arrowheads) is shown terminating in the MTNd (arrow), while the AOT-SF (black arrowhead) can be seen entering the MTNv (white arrowhead). Rostral is to the left. Lower panels, enlargements of the boxed region in the upper panels. AOT-IF labeled by CTB is predominantly GFP-positive. Scale bars: A,B (upper panels), 200 µm; B (lower panels), 50 µm.
(7.44 MB TIF)
