# Supplementary figures and images for: Identification of Retinal Ganglion Cells and Their Projections Involved in Central Transmission of Information about Upward and Downward Image Motion
Source: PLoS One. 2009 Jan 29;4(1):e4320. doi: 10.1371/journal.pone.0004320 (PMC2629575; doi:10.1371/journal.pone.0004320)

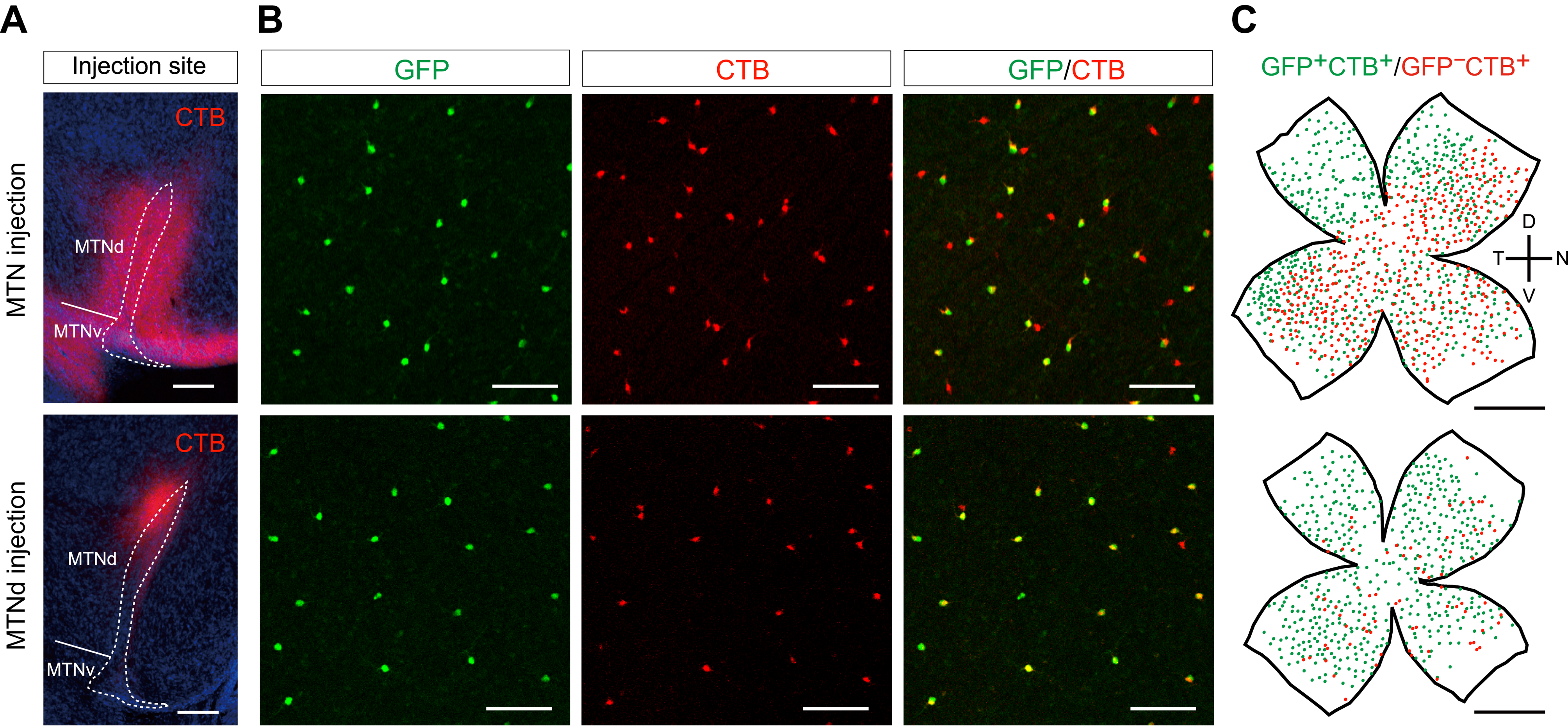

Supplement: Figure S1 — Projection sites of upward- and downward-preferring subtypes are segregated in the MTN. A, Examples of injection of the retrograde tracer CTB-Alexa 555. The whole of the MTN (upper) or the MTNd (lower) received an injection. B, Retrogradely labeled retinal ganglion cells in the pan-ventronasal domain of contralateral retinas at P6. C, Representative drawings of retrogradely labeled cells in the contralateral retinas at P6. CTB+ cells (MTN-P cells) are subdivided into two populations; GFP+CTB+ cells (green dots) and GFP−CTB+ cells (red dots). The number of GFP+CTB+ cells and GFP−CTB+ cells in the pan-ventronasal domain was: 1143 and 1250 (summed over 3 retinas), respectively, when the whole area of the MTN was injected; 708 and 170 (summed over 2 retinas), respectively, when the MTNd region was injected. This result indicates that projection sites of GFP+CTB+ cells and GFP−CTB+ cells are significantly segregated along the dorsoventral axis of the MTN (χ2 = 282.6; p<0.0001). Scale bars: A, 200 µm; B, 100 {lwoer case mu}m; C, 1 mm. (7.18 MB TIF) [file pone.0004320.s001.tif]

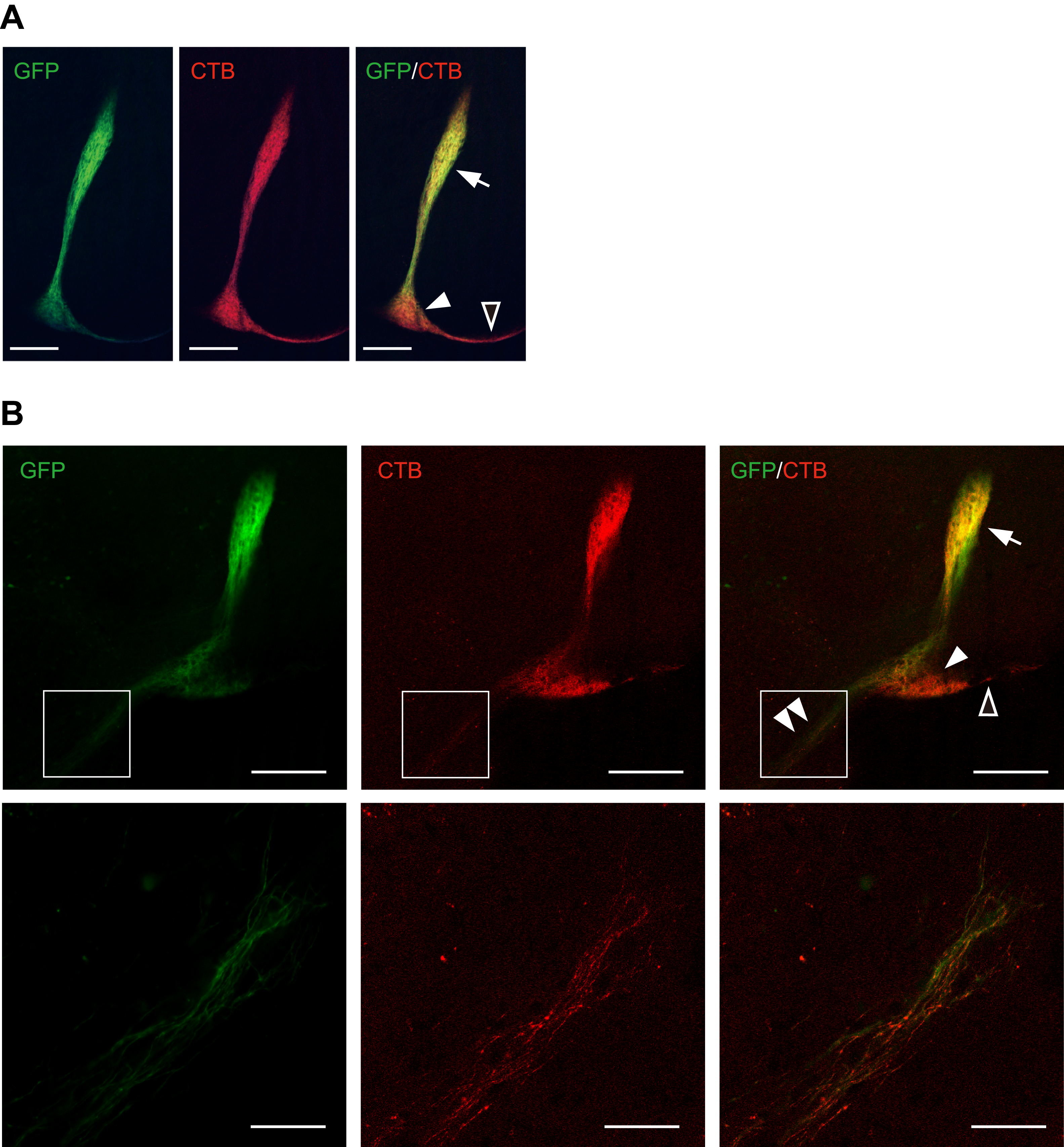

Supplement: Figure S2 — MTN receives retinal axons from AOT-IF and AOT-SF. All retinal ganglion cell axons from an eye had been labeled anterogradely by CTB-Alexa 555 one day before the mice were killed (n = 3 mice for each coronal and parasagittal section). A, Coronal section at P6. The superficially lying AOT-SF (black arrowhead) is entering the MTNv (white arrowhead) to terminate in this region. AOT-SF and MTNv show weaker GFP signals than the MTNd (arrow). B, Parasagittal section at P6. Upper panels, the AOT-IF (double arrowheads) is shown terminating in the MTNd (arrow), while the AOT-SF (black arrowhead) can be seen entering the MTNv (white arrowhead). Rostral is to the left. Lower panels, enlargements of the boxed region in the upper panels. AOT-IF labeled by CTB is predominantly GFP-positive. Scale bars: A,B (upper panels), 200 µm; B (lower panels), 50 µm. (7.44 MB TIF) [file pone.0004320.s002.tif]
